# *Pereskia sacharosa* Griseb. (Cactaceae) Prevents Lipopolysaccharide-Induced Neuroinflammation in Rodents via Down-Regulating TLR4/CD14 Pathway and GABAA γ2 Activity

**DOI:** 10.3390/cimb46070411

**Published:** 2024-07-03

**Authors:** María Fernanda Prado-Fernández, Víctor Manuel Magdaleno-Madrigal, Emmanuel Cabañas-García, Samuel Mucio-Ramírez, Salvador Almazán-Alvarado, Eugenio Pérez-Molphe-Balch, Yenny Adriana Gómez-Aguirre, Edith Sánchez-Jaramillo

**Affiliations:** 1 Departamento de Química, Centro de Ciencias Básicas, Universidad Autónoma de Aguascalientes, Av. Universidad 940, Ciudad Universitaria, Aguascalientes 20131, Aguascalientes, Mexico; fernanda.prado.6696@gmail.com (M.F.P.-F.); eperezmb@correo.uaa.mx (E.P.-M.-B.); 2 Laboratorio de Neuromodulación Experimental, Dirección de Investigaciones en Neurociencias, Instituto Nacional de Psiquiatría Ramón de la Fuente Muñiz, Calz. México Xochimilco No. 101, Col. San Lorenzo Huipulco, Ciudad de México 14370, Mexico; maleno@inprfm.gob.mx; 3 Centro de Estudios Científicos y Tecnológicos No. 18, Instituto Politécnico Nacional, Blvd. del Bote 202 Cerro del Gato Ejido La Escondida, Col. Ciudad Administrativa, Zacatecas 98160, Zacatecas, Mexico; emmanuel.cabanasg@gmail.com; 4 Departamento de Neuromorfología Funcional, Dirección de Investigaciones en Neurociencias, Instituto Nacional de Psiquiatría Ramón de la Fuente Muñiz, Calz. México Xochimilco No. 101, Col. San Lorenzo Huipulco, Ciudad de México 14370, Mexico; mucios@yahoo.com; 5 Laboratorio de Neurofisiología del Control y la Regulación, Dirección de Investigaciones en Neurociencias, Instituto Nacional de Psiquiatría Ramón de la Fuente Muñiz, Calz. México Xochimilco No. 101, Col. San Lorenzo Huipulco, Ciudad de México 14370, Mexico; salmazan@gmail.com; 6 CONAHCyT Research Fellow, Universidad Autónoma de Aguascalientes, Av. Universidad 940, Ciudad Universitaria, Aguascalientes 20131, Aguascalientes, Mexico; 7 Laboratorio de Neuroendocrinología Molecular, Dirección de Investigaciones en Neurociencias, Instituto Nacional de Psiquiatría Ramón de la Fuente Muñiz, Calz. México-Xochimilco 101, Col. San Lorenzo, Huipulco, Ciudad de México 14370, Mexico

**Keywords:** phenolic compounds, LPS, theta oscillation, GABA_A_ γ2, c-fos, CD14 mRNA

## Abstract

*Pereskia sacharosa* Griseb. is a plant used in traditional herbal medicine to treat inflammation. We analyzed the phenolic content of *P. sacharosa* leaves (EEPs) by liquid chromatography–tandem mass spectrometry (LC-MS/MS) and investigated the anti-inflammatory properties of EEPs and its flavonoid fraction (F10) in animal models subjected to acute neuroinflammation induced by bacterial lipopolysaccharide (LPS). Coronal brain sections of C57BL/6JN male mice or Wistar male rats administered with EEPs or F10 before LPS were subjected to in situ hybridization to determine c-fos and CD14 mRNA levels in the hypothalamus or GABA_A_ γ2 mRNA levels in the hippocampus. Theta oscillations were recorded every 6 h in the hippocampus of Wistar rats. In total, five flavonoids and eight phenolic acids were identified and quantified in *P. sacharosa* leaves. Either EEPs or F10 crossed the blood–brain barrier (BBB) into the brain and reduced the mRNA expression of c-fos, CD14, and GABA_A_ γ2. A decrease in theta oscillation was observed in the hippocampus of the LPS group, while the F10 + LPS group overrode the LPS effect on theta activity. We conclude that the bioactive compounds of *P. sacharosa* reduce the central response to inflammation, allowing the early return of ambulatory activity and well-being of the animal.

## 1. Introduction

The interconnection between ethnopharmacology and integrative medicine may serve to discover molecules with potential therapeutic properties. Studies have demonstrated investigations of traditional medicine, inspiring approaches for drug discovery and biodiversity conservation [1].

Since new treatments are being sought for various neurodegenerative diseases, including Alzheimer’s disease, Parkinson’s disease, sleep–wake disorders, and stroke, where chronic neuroinflammatory responses are involved, research on medicinal plants represents an alternative to treat the high incidence of inflammatory diseases that affect the central nervous system (CNS) [2].

The biological activities of medicinal plants are associated with their phytochemical profile since they produce various secondary metabolites including phenolics, flavonoids, alkaloids, terpenes, saponins, and sterols. These bioactive compounds can provide health benefits, which justifies their use and isolation as therapeutic agents with essential applications in modern medicine [3]. The species of the genus *Pereskia* are used in traditional medicine due to their anti-inflammatory, antioxidant, analgesic, cytotoxic, and antimicrobial properties, as well as their antisyphilitic, expectorant, and emollient properties [4]. This genus is distributed from México to South America, being more abundant in Brazil, Bolivia, Paraguay, and Argentina [5]; *P. sacharosa* leaves and fruits are used in traditional medicine to treat skin disorders and pain, while the thorns are used to treat muscle pain [6]. In addition, in a survey performed by [7], they found that *P. sacharosa* appears among the traditionally used plants in Bolivia for treating children with dehydration, skin disorders, and musculo-skeletal pain, indicating their applications for pain relief, similar to that reported by [8] in Brazil. For closely related plant species such as *P. bleo* [9] and *P. aculeata* [10], evidence suggests that they are a rich source of phenolic and antioxidant metabolites with functional properties. In this regard, in previous reports, we demonstrated that the phenolic metabolite kaempferol and other secondary metabolites of *P. sacharosa* are highly hydrophilic and can cross the BBB, laying along the lateral borders of the third ventricle [11]; nevertheless, although this plant species is traditionally used for its pain relief and anti-inflammatory properties, evidence validating the functional properties is scarce.

In this research, we evaluated the anti-inflammatory activity of *P. sacharosa* in a neuronal population of the mediobasal hypothalamus (MBH) that controls energy metabolism during illness. We also evaluated the theta oscillation and GABAergic activity of the hippocampus in rodents subjected to neuroinflammation with LPS. In addition, the analysis of phenolic compounds in *P. sacharosa* leaves by LC-MS/MS allowed us to identify metabolites with biological activity that might be used as an alternative treatment for inflammatory diseases, especially those that affect the CNS.

## 2. Materials and Methods

### 2.1. Plant Material

Healthy leaves of *P. sacharosa* (identified by Biologist Julio Martínez Ramírez, voucher No. 18,471, UAA Herbarium) were collected every 15 days before 9 a.m. for 8 months (April–December 2022) at the Universidad Autónoma de Aguascalientes, Mexico. The collected leaves were dried at 40 °C in an oven (Torrero originales^®^, Aguascalientes, Mexico), and the dried material was then pulverized in a mortar [164.17 g dry weight (DW)] and stored in dark conditions at room temperature until analysis.

#### 2.1.1. LC-MS/MS Multiple Reaction Monitoring (MRM) Assay of Polyphenols

An aliquot of 50 mg of *P. sacharosa* leaves was extracted using 100% methanol spiked with an internal standard GR24 and analyzed using LC-MS/MS as previously described in Vu and Alvarez [12], with some modifications. Briefly, 900 µL of 100% methanol was added. Then, two stainless steel beads (SSB 32) were incorporated into the mixture to help with the disruption and homogenization process performed in a TissueLyserII (Qiagen, Hilden, Germany) operated at 10 Hz for 5 min. The disrupted samples were centrifugated at 16,000× *g*, and the supernatants were carefully recovered. The resulting pellet was also recovered and subjected to a subsequent extraction cycle using the same procedure. The recovered supernatants were combined and vacuum-dried using a SAVANT speed-vac (Thermo Scientific™, Waltham, MA, USA). The chromatographic and mass spectrometric analysis were carried out using a Shimadzu Nexera II HPLC system (Shimadzu Scientific Instruments INC, Columbia, MD, USA) hyphenated with a Sciex QTRAP 6500+ mass spectrometer (AB Sciex Pte. Ltd., Framingham, MA, USA). The compounds of interest were subjected to chromatographic separation in a ZORBAX Eclipse XDB C18 column (2.1 mm × 100 mm, Agilent, Santa Clara, CA, USA). The elution gradient program consisted of a mixture of 2% acetic acid (A) and 100% acetonitrile (B) as follows: 6% B for 1 min, to 17% B in 4 min, to 20% B in 3 min, to 90% B in 8 min, hold at 90% B for 2 min, to 6% B in 1 min. The flow rate was set at 0.4 mL/min.

To control sample acquisition and data analysis, Analyst software (version 1.6.3) was employed. In addition, for tuning and calibrating the QTRAP 6500+ mass spectrometer, we followed the manufacturer’s recommendations. On the other hand, the target compounds were detected using MRM transitions optimized using standards. For quantification purposes, an external standard curve was prepared using a series of standards containing different concentrations of compounds and fixed concentrations of the internal standards mixture. The following compounds were included in the assay: apigenin, caffeic acid, catechin, naringenin chalcone, chlorogenic acid, cinnamic acid, cyanidin, daidzein, delphinidin, epicatechin, ferulic acid, gallic acid, genistein, hesperetin, kaempferol, luteolin, naringenin, p-coumaric acid, phloretin, proanthocyanidin A2, procyanidin B2, protocatechuic acid, quercetin, quercetin-3-galactoside, quercetin-3-glucoside, resveratrol, rutin, syringic acid, and vanillic acid.

#### 2.1.2. Preparation of Extract and Fractionation

The ethanolic extract of *P. sacharosa* (EEPs) and fraction 10 (F10) were obtained following the methodology reported by Ruiz-Velasco-Martínez et al. [11]. Hence, a successive maceration extraction process was carried out using hexane, chloroform, ethanol, methanol (JT Baker^®^, Phillipsburg, NJ, USA), and 112 g of dry leaves in a 1:2 mass/volume ratio. Each extraction process was performed for 24 h, under constant agitation (80 rpm) and in dark conditions. The extracts were filtered through a qualitative-grade filter paper (Ahlstrom grade 54, Pottsville, PA, USA) and vacuum-dried at 40 °C in a rotary evaporator (model D-402-10, Sev-Prendo, Puebla, Mexico). The EEPs (1.5 g) was fractionated by open-column chromatography (height: 50 cm, diameter: 4.5 cm, and volume: 500 mL) with 60 g of silica gel 60 (0.40–0.063 mm, 230–400 mesh, Merck, Boston, MA, USA). The separation process was conducted using a gradient elution system comprising mixtures of chloroform/methanol (100:0, 95:5, 90:10, 85:15, 80:20, 70:30, 60:40, 50:50, and 0:100). By using this separation process and collecting fractions every 25 mL, a total of 130 samples were collected and categorized in 26 pools based on their chemical similarity observed using thin-layer chromatography (TLC). For monitoring purposes, TLC was performed on aluminum plates coated with silica gel 60 F254 (Merck, Darmstadt, Germany). TLC was observed under an ultraviolet light lamp (UV Light, Spectronics Co., Melville, NY, USA) at 254 nm and 365 nm. Subsequently, TLC was sprayed with a solution of 1% methanolic 2-amino-ethyl diphenyl boric acid (NP, Sigma-Aldrich^®^ Co., Munich, Germany) and 5% polyethylene glycol (PEG, Sigma-Aldrich^®^ Co., St. Louis, MO, USA). TLC was visualized under UV light at 365 nm. The fraction F10 (120 mg) was like C1F9 [11]. EEPs and F10 were then taken to evaluate the anti-inflammatory activity.

### 2.2. Anti-Inflammatory Activity of P. sacharosa

#### Animals

Adult male Wistar rats (n = 10) from the vivarium of Instituto Nacional de Psiquiatría Ramón de la Fuente Muñiz (INPRFM), 8 weeks old, and weighing 270–320 g and adult male mice C57BL6/JN (n = 36) from the vivarium of Instituto de Biotecnología UNAM, 10 weeks old, and weighing 28–30 g were used in this study. The animals were acclimatized to standard environmental conditions (lights on between 07:00 and 19:00 h, room temperature 22 °C ± 1 °C, rat chow 2918 from Envigo (Indianapolis, IN, USA) or mice chow 2018SX from Harlan (New York, NY, USA), and water ad libitum). Protocols followed the NIH guide for the care and use of laboratory animals (8th ed.), and the Official Mexican Norm for the production, care, and use of laboratory animals (NOM-062-ZOO-1999). All experimental protocols were reviewed and approved by the Animal Ethics and Scientific Committee of INPRFM (INP-NC19110.0, INP NC12.3140.1), following the recommendations of the Research and Ethics Committee of the International Association for the Study of Pain. Efforts were made to minimize the number of animals and their suffering.

### 2.3. Experimental Design

All treatments were administered intraperitoneally (i.p.). LPS (Sigma O127:B8; Sigma Chemical Co., St. Louis, MO, USA) doses for mice and rats were 150 µg/kg body weight (BW) and 250 µg/kg BW, respectively. It has been previously reported that these doses are sufficient to activate the IL-1β and IL-6 proinflammatory response in the periphery, in parallel with a decrease in the expression of thyroid hormone transporters in the blood–brain barrier (BBB) and a food intake decrease in the hypothalamic arcuate nucleus, both in C57/BL6 mice and Sprague Dawley rats [13,14,15]. The LPS doses used in this study were far from those used to cause severe inflammation and cognitive impairment [16], as the animal’s well-being returned to baseline 12 h later [14,17].

The dose of the EEPs and F10 was 30 mg/kg BW for both species. The vehicle group consisted of an isotonic saline solution (ISS) + Tween 80 [ISS + 50 μL of tween 80; Sigma-Aldrich^®^ Co., St. Louis, MO, USA]. All doses were dissolved in 100 µL of vehicle per mouse and 250 µL per rat.

In situ hybridization was performed in 30 mice as follows: vehicle (n = 5), LPS 3 h (n = 5), EEPs + LPS 3 h (n = 5), F10 + LPS 3 h (n = 5), LPS 9 h (n = 5), and EEPs + LPS 9 h (n = 5) groups. Six additional mice, vehicle (n = 3) and F10 + LPS (n = 3), were sectioned separately to corroborate the presence of F10 in the brain. In addition, adult Wistar male rats were assigned to the LPS (n = 5) or F10 + LPS (n = 5) groups and underwent hippocampal electroencephalography (EEG) for 6 h.

### 2.4. Sacrifice and Tissue Processing

At the end of each treatment, animals were overdosed with pentobarbital (50 mg/kg BW, i.p.; Pet’s Pharma) and decapitated with a sharp guillotine (rats) or with dissection scissors (mice); the brains were removed, frozen on dry ice, and then stored at −80 °C until use. Serial 18 μm coronal brain sections from mice tissue were collected every six sections rostro-caudally from bregma −1.46 to −2.06 mm [18], using a Leica cryostat (Leica CM3050 S, Nussloch GmbH, Nussloch, Germany). The sections contained the rostro-medial part of the hypothalamic arcuate nuclei and the whole dorsal hippocampus. The slices were thaw-mounted on Fisherbrand Superfrost Plus Microscope Slides (Thermo Fisher Scientific, Waltham, MA, USA; Cat #12-550-15), air-dried, and stored at −80 °C until use. Some sections were stained with Nissl to identify and collect the regions of interest.

### 2.5. F10 Presence in the Mouse Brain

A group of six mice was divided into two groups: a vehicle group (n = 3), administered only with ISS + Tween 80, and an F10 + LPS group (n = 3), administered with F10 (30 mg/kg BW) 30 min before LPS administration. After a period of 3 h after monitoring, mice were deeply anesthetized with pentobarbital (50 mg/kg BW, Pet’s Pharma) and decapitated, and their brains were dissected, frozen on dry ice, and stored at −80 °C until use. Each brain was cut into 25 μm sections on a cryostat from bregma −1.46 to −3.4 mm [18] to determine the presence of F10 compounds in the brain. Duplicates with six rostro-caudal sections were collected on Fisherbrand Superfrost Plus Microscope Slides (Thermo Fisher Scientific; Cat. 12-550-15). Thawed sections were observed directly under epifluorescence light microscopy without a coverslip or any mounting medium that could mask the fluorescence generated by the F10. Fluorescent signals were recorded with a Leica DM 1000 LED fluorescence microscope, using an HI PLAN 10×/0.25, HI PLAN 20×/0.40, or 40×/0.65 objective, with bandpass filter sets of 340–380 nm/dichromatic mirror 400 nm/barrier low pass 425 nm; 480/40 nm/dichromatic mirror 505 nm/barrier bandpass filter 527/30 nm; and 560/40 nm/dichromatic mirror 595 nm/barrier bandpass filter 645/75 nm (Leica Microsystems AG, Wetzlar, Germany). The images were captured using a digital Firewire camera (DFC450C, Leica) and Leica Application Suite X, version 2.0.0 software. Sections were exposed to each bandpass filter set by switching the filter sets, and the resulting images were processed in Adobe^®^ Photoshop CS6, version 13.0 x64 (Adobe Systems, Inc., San Jose, CA, USA) software to create a composite image of each analyzed brain.

### 2.6. cRNA Probes for In Situ Hybridization

cRNA probes for in situ hybridization were generated as previously described [19]. The mouse CD14 riboprobe was generated from the full-length cDNA, cloned in a pRc/CMV vector 5.4 kb (Sigma-Aldrich Cat. E8022). The mouse GABA_A_ γ2 subunit receptor, transcript variant 1 (810 bp long, corresponding to 172–982 of NM_008073) was cloned in a pGEM-T Easy vector (Promega, Madison, WI, USA, Cat. V012473). A cRNA probe for c-fos was generated from a 531 pb fragment, spanning the 134–665 rat cDNA sequence as previously described [20].

### 2.7. Fluorescent In Situ Hybridization (FISH)

In situ hybridization was performed as previously described [19]. Briefly, two adjacent series (S1, S2) of 18 μm thick coronal sections (n = 5 animals per group), each containing every six slices, were hybridized either with a UTP-digoxigenin (dig)-labeled c-fos and a UTP-fluorescein-labeled CD14 riboprobe (S1 series) or with a UTP-dig-labeled GABA_A_ γ2 riboprobe (S2 series). The S1 and S2 series were fixed in 4% paraformaldehyde −0.1 M PB (pH 7.4) for 10 min, rinsed in PBS for 5 min, acetylated with 0.25% acetic anhydride in 0.1 M triethanolamine for 10 min, dehydrated through graded concentrations of alcohol (70%, 80%, 95%, 100%; 2 min each) and chloroform (10 min), partially rehydrated in 95% ethanol, and then processed for in situ hybridization. Two microliters of dig-c-fos and 2 μL of fluorescein-CD14 riboprobes (S1 series) or 2 μL of dig-GABA_A_ γ2 riboprobe (S2 series) were added to an 80 μL hybridization buffer (50% formamide, 2× standard saline citrate [SSC], 1× Denhardt’s, 10 mM dithiothreitol [DTT], 250 μg/mL of single-stranded salmon sperm DNA, 10% dextran sulfate, 0.5% sodium dodecyl sulfate [SDS]), spotted on each slide, sealed under a parafilm coverslip, and incubated at 54 °C overnight. Coverslips were then removed, and the slides were rinsed in 1× SSC (0.15 M NaCl, 0.015 M NaCl) for 15 min at room temperature, digested by RNase A (25 μg/mL; Sigma, Cat. R-4875) for 1 h at 37 °C, and rinsed in 1× SSC for 15 min at room temperature, 0.5× SSC for 15 min at 65 °C, and 0.1× SSC twice for 30 min at 65 °C. The specificity of hybridization for each antisense probe has been verified elsewhere using sense probes, which resulted in the absence of a specific hybridization signal in the tissues of interest [20].

The UTP-dig c-fos and UTP-dig GABA_A_ γ2 probes were detected with a peroxidase-conjugated, anti-digoxigenin antibody (diluted 1:100 in 1% blocking reagent, Roche, Basel, Switzerland; Cat.11096176001). The dig-UTP signal was amplified with the Tyramide Signal Amplification (TSA) Plus Biotin Kit (Cat# NEL749B001KT, Perkin Elmer, Waltham, MA, USA) for 30 min, using the biotin amplification reagent at 1:100 dilution in 0.05 M Tris (pH 7.5) containing 0.01% H_2_O_2_. The UTP-fluorescein CD14 signal was detected with a monoclonal antibody to fluorescein from mouse–mouse hybrid cells (Roche, Cat. 11426320001, diluted 1:100 in 1% blocking reagent). The biotin deposits and the anti-fluorescein-antibody signals were detected with Alexa Fluor 488-conjugated Streptavidin and Cy3-conjugated anti-mouse IgG (Jackson Immunoresearch, West Grove, PA, USA; 1:200), respectively. Sections were rinsed thoroughly in PBS and Tris 0.05 M (pH 7.5), and coverslipped with a Vectashield antifade mounting medium with DAPI (Vector Laboratories, Newark, CA, USA, Cat H-2000). Immunofluorescence imaging and data analysis were performed using epifluorescence light microscopy and confocal analyses as described below.

### 2.8. Immunofluorescence Imaging and Data Analysis

Immunofluorescence sections containing c-fos/CD14 mRNA signals (S1 series) or GABA_A_ γ2 signals (S2 series) were observed using a Leica DM 1000 LED fluorescence microscope with an HI PLAN 10×/0.25 or 40×/0.65 objective, with bandpass (BP) filter sets of 340–380/dichromatic mirror (DM) 400/barrier low pass (LP) 425; BP 480/40/DM 505/barrier BP 527/30; and BP 560/40/DM 595/barrier BP 645/75 for DAPI, Alexa 488, and Cy3, respectively. Images were captured using a digital Firewire camera (DFC450C, Leica) and Leica Application Suite software. Sections were double-exposed while switching filter sets for each fluorochrome and superimposed in Photoshop CS6 (Adobe Systems, Inc.) using an iMac computer to create a composite image of the same field.

S1 series containing c-fos+/CD14+ cells from the hypothalamic arcuate nuclei, with evident nuclei staining (DAPI+), were considered and counted. To avoid counting the same nuclei more than once, we overlaid a grid with square sections (24.5 μm × 24.5 μm) on the images and counted the DAPI+ nuclei marked with an x. Image-J software v1.52k with a plugin analyzer-cell counter was used for the analyses (v 1.44p, National Institute of Health, available at https://imagej.net/software/imagej/ (accessed on 5 June 2024)).

### 2.9. Confocal Imaging and Data Analysis

Slides containing a GABA_A_ γ2 mRNA signal in the dorsal hippocampus (S2 series) were acquired with a Zeiss 900 laser scanning confocal microscope with Airyscan 2, equipped with a 488 nm diode laser (Alexa Fluor 488 dye), attached to an Axio Observer.Z1/7 microscope and a 10× Plan Apochromat 10×/0.45 objective (Carl Zeiss AG, Oberkochen, Germany).

Images from each section (18 µm thickness) were acquired bilaterally on the optimal focal plane with the 488 nm diode laser, with a pinhole diameter of 1 airy unit and detector gain. Laser power was adjusted to provide an optimal dynamic range for the measurements (the same in all slides). The entire area of interest was scanned (approx. + 46 images) and merged into a single panoramic image. Images were then analyzed for integrated optical density (IOD) using computer-assisted densitometry software (Image Pro Plus 4.5, Media Cybernetics, Inc., Rockville, MD, USA). Images were converted to a grayscale (0 (black)–255 (white)), and their background was subtracted. Subsequently, the IOD for GABA_A_ γ2+ cells was quantified in the hippocampus. Shortly after, left and right hemisphere areas were manually outlined in at least six sections per mice. IOD values (density × area) were determined per section. The IOD is reported in arbitrary units. The average IOD was obtained for each region of interest (bilateral hippocampus) by pooling IOD values of mice from each experimental group.

Representative images from each experimental group were projected in 2.5D with the Zen software (Blue Edition v 3.4) from Zeiss, in which the angles of the X-, Y-, and Z-axes were manually moved to obtain a frontal view image saved in a 300 dpi .tiff format.

### 2.10. Statistical Analysis

Results are reported as mean ± SEM (standard error mean) per group. Statistical analysis was performed using SigmaPlot v12.3 (Systat Sofware, Inc., Palo Alto, CA, USA) and Graph Pad Prism [version 10.1.0 (264)]. Statistical significance among groups was determined by one-way ANOVA and the multiple comparison tests (Dunnett’s, Fisher where stated), where data met normality and homogeneity of variance assumptions. Differences were considered significant at *p* < 0.05.

### 2.11. Theta Oscillation in the Hippocampus in Freely Moving Rats

Ten rats underwent stereotaxic surgery. They were anesthetized with ketamine chloride (50 mg/kg BW) and xylazine chloride (15 mg/kg BW, i.p.) and placed in a stereotaxic apparatus with their bregma and lambda in the horizontal plane. An incision was made in the skin covering the skull to expose it and determine the position of the electrodes; two holes (1 mm) were drilled, and then the stainless steel electrodes (0.300 mm diameter) were implanted in both the left and right dorsal hippocampus (AP, −3.2 mm; L, +2.7 mm; H, +3.2 mm), as reported previously [21]. In addition, another electrode was connected as the ground to the parietal bone. The electrodes were fixed to the skull with dental acrylic. After surgery, animals were treated with analgesic (Butorphanol, 0.4 mg/kg) and antibiotic (Amoxicillin, 0.6 mg/kg). After one week of post-surgical recovery, the animals were randomly assigned to two experimental groups: the LPS group (n = 5), which received LPS (250 µg/kg BW), and the F10+ LPS group (n = 5), which received F10 (30 mg/kg BW) 30 min before receiving a dose of LPS (250 µg/kg BW).

The theta oscillation in the hippocampus was recorded for 6 h, using a polygraph model 7 with amplifier P511 (Grass, Boston, MA, USA). The signals were amplified, bandpass-filtered between 1 and 100 Hz, and digitized (500 samples/s) with a homemade A/D system developed in our laboratory [22]. All digitized data were stored in an optical disk. Offline spectral analyses using Fast Fourier Transform (FFT) methods during periods of 20 s every 60 min (baseline, LPS, F10, and posttreatment) were performed with a MATLAB custom routine (2019a, The Mathworks Inc., Natick, MA, USA). Power numerical values corresponding to the theta bandwidth were normalized as 100% against the maximum power of baseline (4–12 Hz). Time–frequency domain spectrograms from 20 s epochs were obtained by the same means (95% overlapping 2.5 s Hamming windows). Finally, the coherence analysis to measure the phase consistency between pairs of recordings for theta oscillation was performed.

## 3. Results

### 3.1. Phenolic Acid, Flavonoid Identification, and Contents in P. sacharosa Extract

The identification and quantitation of phenolic metabolites present in *P. sacharosa* leaves were performed using LC-MS/MS. In this study, the phytochemical profile of methanolic extract was compared with 28 authentic analytical standards and then quantified. A total of 13 phenolic compounds belonging to different classes, such as phenolic acids and flavonoids, were detected (Table 1). As previously reported by our group [11], we found the flavonoid kaempferol as one of the constituents of *P. sacharosa*; when this metabolite was quantified, a total of 66.6 ng/g was found. In addition, other flavonoids such as naringenin (4.5 ng/g), glycosylated flavonoids such as quercetin galactoside (482.88 ng/g), and quercetin glucoside (5.49 ng/g) were also observed. On the other hand, phenolic acids were present in higher concentrations than flavonoids. In this regard, eight phenolic acids were identified, including cinnamic acid (2803.64 ng/g), *p*-coumaric acid (2637.72 ng/g), ferulic acid (1609.37 ng/g), vanillic acid (1237.25 ng/g), caffeic acid (564.78 ng/g), syringic acid (388.83 ng/g), chlorogenic acid (271.02 ng/g), and gallic acid (9.92 ng/g).

### 3.2. Presence of F10 in the Mouse Brain

**Figure 1:**
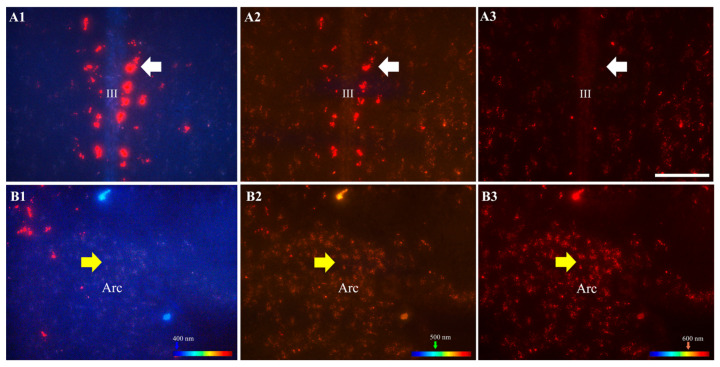
Presence of F10 in the mouse brain. Adult male mice received 30 mg/kg of the purified fraction F10 of *P. sacharosa* i.p., 30 min before the administration of 150 μg/kg of LPS i.p. Brain sections were cut at 25 μm of thickness, stored at −80 °C, and thawed to be observed under an epifluorescence light microscope with a filter for DAPI (UV excitation filter 340–380 nm, emission filter 425 nm). (**A1**,**B1**) An excitation filter 480/40 nm and emission filter 527/30 nm; (**A2**,**B2**) an excitation filter 560/40 nm and emission filter 645/75 nm (**A3**,**B3**). Photomicrographs of representative coronal brain sections around bregma −2.16 mm show the migration of F10 along the third ventricle (III; white arrows in **A1**–**A3**) and the hypothalamic arcuate nucleus (Arc; yellow arrows in (**B1**–**B3**)), 3 h after LPS administration. The fluorescence for F10 aligned around the III ventricle is brighter and larger (**A1**,**A2**) than the small particles observed in the arcuate, which have a wider emission range (**B1**–**B3**). Scale bar = 50 μm.

The migration of F10 to the brain was corroborated in a set of sections of the F10 + LPS 3 h group using an epifluorescence light microscope (Figure 1; bregma −2.16 mm). Self-fluorescence F10 particles crossed the BBB and migrated along the walls of the third ventricle (III), as reported by Ruiz-Velasco-Martínez et al. [11]. Self-fluorescence F10 particles were brighter and more prominent along the third ventricle (Figure 1A1–A3) than the smaller F10 particles observed in the hypothalamic arcuate nucleus area (Figure 1B1–B3). The brighter and larger red fluorescence for F10 was visible with the DAPI and green filters (emissions at 425 nm and 527 nm, respectively; Figure 1A1,A2), while the pink light-red fluorescence for F10 was visible with all the filters used (emission at 425–625 nm; Figure 1B1–B3).

### 3.3. Analysis of C-Fos and CD14 mRNA + Cells in the Arcuate Nucleus of Mice

**Figure 2:**
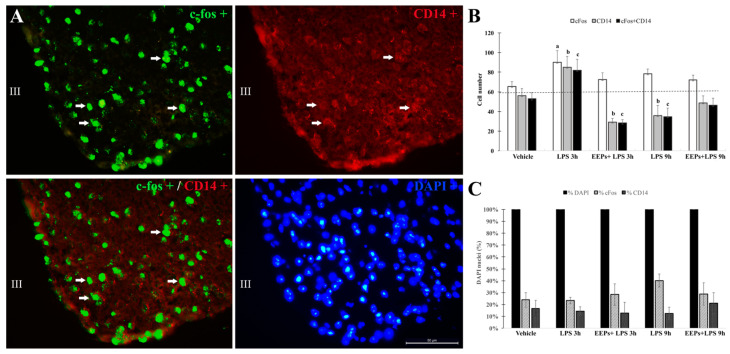
Response of c-fos and CD14 mRNA in the arcuate nucleus of mice receiving *P. sacharosa* extract (EEPs) before LPS. Dual-fluorescent in situ analyses for c-fos+/CD14+ cells were made under an epifluorescence light microscope to count DAPI+ nuclei (blue fluorescence; UV excitation 340–380 nm/emission 425 nm), c-fos+ cells (green fluorescence; excitation filter 480/40 nm/emission 527/30 nm), and CD14+ cells (red fluorescence; excitation filter 560/40 nm/emission 645/75 nm), at bregma −1.46 to −2.06 mm. (**A**) Fluorescently labeled c-fos+ mRNA and CD14+ mRNA cells in the hypothalamic arcuate nucleus of male mice exposed to LPS 3 h (white arrows in (**A**) denote some examples of double labelled cells). (**B**) Dual-labeled c-fos+/CD14+ cells increase in the LPS 3 h group and decrease below basal levels in EEPs+ LPS 3 h and LPS 9 h groups; a dotted line from the vehicle to the experimental groups highlights this difference. (**C**) Percentage of c-fos+ and CD14+ cells regarding DAPI+ nuclei (blue fluorescence). a: Significantly different from the vehicle (*p* < 0.05); b, c: significantly different from the vehicle, EEPs + LPS 3 h, and LPS 9 h (*p* < 0.05). III = third ventricle. Scale bar = 50 μm.

Dual-fluorescent in situ (FISH) analyses were made on images taken from the hypothalamic arcuate nucleus (Figure 2A; bregma −2.16 mm), with an epifluorescence light microscope to count DAPI+ nuclei, c-fos+, and CD14+ cells (bregma −1.46 to −2.06 mm). c-fos and CD14 mRNA + cells were observed in all the experimental groups (Figure 2B). c-fos mRNA + cells increased significantly in the LPS 3 h group [vehicle 68 ± 4 vs. LPS 3 h 93 ± 5; *p* = 0.0027] but did not change in any of the other experimental conditions [vehicle 68 ± 4 vs. EEPs + LPS 3 h 72 ± 7 (*p* = 0.327); vehicle 68 ± 4 vs. LPS 9 h 83 ± 2 (*p* = 0.089); vehicle 68 ± 4 vs. EEPs + LPS 9 h 72 ± 5 (*p* = 0.90)].

All the CD14 mRNA+ cells co-expressed c-fos mRNA in the arcuate (Figure 2B). Compared to the vehicle, double-labeled cells increased significantly in the LPS 3 h group [vehicle 54 ± 4 vs. LPS 3 h 82 ± 4 (*p* = 0.0008)], decreased below the vehicle values in the EEPs + LPS 3 h and LPS 9 h groups [vehicle 54 ± 4 vs. EEPs + LPS 3 h 28 ± 4 (*p* = 0.0004); vehicle 54 ± 4 vs. LPS 9 h 37 ± 2 (*p* = 0.034)], and did not change in the EEPs + LPS 9 h group [vehicle 54 ± 4 vs. EEPs + LPS 9 h 46 ± 7 (*p* = 0.532)]. Statistical significance among groups was determined by one-way ANOVA and Dunnett’s multiple comparison test.

Thirty percent of all the nuclei stained with DAPI (100%) corresponded to c-fos+ mRNA cells in the arcuate. However, fifteen percent or less corresponded to CD14 (Figure 2C).

### 3.4. Analysis of GABA_A_ γ2 mRNA Expression in the Dorsal Hippocampus of Male Mice

**Figure 3:**
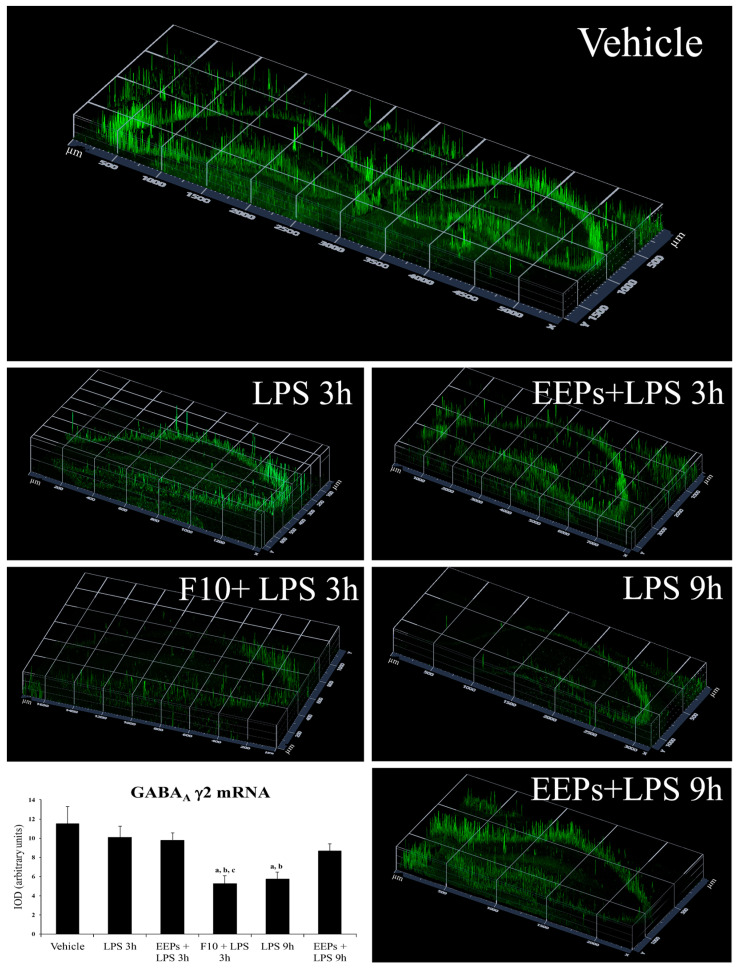
Fluorescent in situ hybridization for GABA_A_ γ2 mRNA in the dorsal hippocampus of C57/BL6/JN male mice exposed to LPS. Representative 2.5D view images were obtained with a Zeiss 900 laser scanning confocal microscope in the vehicle, LPS 3 h, F10 + LPS 3 h, LPS 9 h, and F10 + LPS 9 h groups (Zen Blue Edition software v 3.4, from Zeiss). The immunofluorescence intensity by the FISH technique can be visualized as peaks of different heights in the Z-axis. The highest peaks can be interpreted as exhibiting the highest intensity and content of GABA_A_ γ2. The X- and Y-axes show the length and width in µm of the hippocampal region area scanned. The vehicle shows the highest peaks and staining intensity compared to those of F10 + LPS 3 h or LPS 9 h. Contrary to F10 + LPS 3 h or LPS 9 h, EEPs + LPS 9 h returned to the basal GABA_A_ γ2 mRNA level. The graph at the bottom left shows the semi-quantitative analysis for GABA_A_ γ2 mRNA, expressed as integrated optical density (IOD; density × area = arbitrary units). Statistical significance among groups was determined by one-way ANOVA and the Holm–Sidak post hoc test; differences were considered significant at *p* < 0.05. a: Significantly different from the vehicle (*p* < 0.01); b: significantly different from LPS 3 h (*p* < 0.05); c: significantly different from EEPs+ LPS 3 h (*p* < 0.05).

FISH for GABA_A_ γ2 mRNA in the dorsal hippocampus of C57/BL6/JN male mice exposed to LPS showed a similar mRNA level expression for the LPS 3 h and EEPs+ LPS 3 h groups when compared to the vehicle [LPS 3 h 10.07 ± 1.76 vs. vehicle 11.54 ± 4 (*p* = 0.35); EEPs +LPS 3 h 9.76 ± 0.7 vs. vehicle 11.54 ± 4 (*p* = 0.341); IOD, arbitrary units] (Figure 3). However, there was a significant decrease in GABA_A_ γ2 mRNA expression in the hippocampus of mice receiving F10, 30 min before receiving LPS [LPS 3 h 10.07 ± 1.76 vs. F10 + LPS 3 h 5.25 ± 0.8 (*p* = 0.009); IOD, arbitrary units]. This response was like that observed for the LPS 9 h group [LPS 3 h 10.07 ± 1.76 vs. LPS 9 h 5.72 ± 0.7 (*p* = 0.01); IOD, arbitrary units] (Figure 3). No changes were seen in GABA_A_ γ2 mRNA expression between LPS 3 h and EEPs + LPS 3 h or EEPs + LPS 9 h groups [LPS 3 h 10.07 ± 1.76 vs. EEPs + LPS 3 h 9.76 ± 0.7 (*p* = 0.85); LPS 3 h 10.07 ± 1.76 vs. EEPs + LPS 9 h 8.66 ± 0.7 (*p* = 0.31); IOD, arbitrary units] (Figure 3). Statistical significance among groups was determined by one-way ANOVA and Fisher’s multiple comparison test.

### 3.5. Local Field Potential Time–Frequency Domain and Coherence Analyses in the Rat Brain

**Figure 4:**
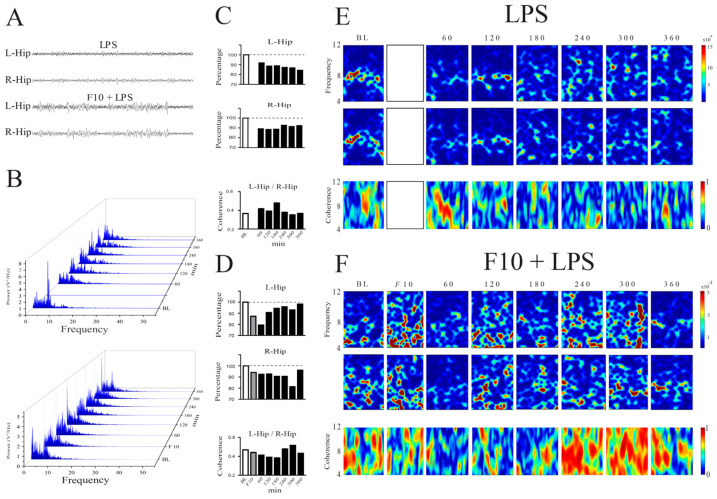
LPS administration decreases theta oscillations in the hippocampus of freely moving rats. (**A**) Representative LFP of the left (L) and right (R) hippocampus in rats administered with LPS or F10 + LPS. (**B**) In the Fast Fourier Transform waterfall plot, a high theta LFP peak can be observed at the baseline, disappearing after the LPS administration, whereas the pretreatment with F10 maintained the theta LFP over time. (**C**) Normalized power spectrum (100%) and normalized coherence (1) of theta LFP of the LPS group. Note the significant decrease in the theta LFP power spectrum. (**D**) Normalized power spectrum (100%) and normalized coherence (1) of the theta LFP of the F10 + LPS group. (**E**) Representative spectrograms of the theta bandwidth and coherence of the LPS group, where the evolution in the time domain is shown. (**F**) Representative spectrograms of the theta bandwidth and coherence of the F10 + LPS group, where the evolution in the time domain is shown.

LPS induced evident signs of sickness almost 60 min after administration; these signs were maintained during the total time of local field potential recording (LFP) (Figure 4A), and after 180 min, all animals behaviorally showed that the disease was established. On the other hand, pretreatment with F10 30 min before LPS administration showed that the signs of sickness were less severe. Power spectrum densities of 0–55 Hz in bandwidth are presented in Figure 4B and divided into 1–4 Hz (delta), 4–12 Hz (theta), 13–30 Hz (beta), and 35–55 Hz (gamma 1) bandwidths. No significant changes were observed except for the theta oscillation, whereas progressive decreases in theta oscillations, power spectrum density in the frequency domain of both the left hippocampus (L-Hip) (F_7,28_ = 3.01, *p* < 0.01) and the right hippocampus (R-Hip) (F_7,28_ = 8.08, *p* < 0.001), and the time domain during 6 h of recording were observed (Figure 4C). In parallel with the effects on the power spectrum, the coherence in both L-Hip and R-Hip pairs showed significant changes (F_7,3200_ = 284.2, *p* < 0.001) (Figure 4D). One remarkable finding was that, although there was a decrease in the frequency domain with the F10 pretreatment (Figure 4E,F), this decrease was not significant in both L-Hip (F_7,32_ = 0.527, *p* = 0.8068) and R-Hip (F_7,32_ = 0.635, *p* = 0.7232). In addition, the coherence was significant (F_7,3200_ = 239.8, *p* < 0.001).

## 4. Discussion

Bioactive compounds in *Pereskia* species, such as alkaloids, tannins, terpenoids, sterols, coumarins, phenolic compounds, flavonoids, saponins, and terpenes, have been reported as therapeutic agents due to their anti-inflammatory, antioxidant, analgesic, cytotoxic, and antimicrobial activities [4,23,24,25,26,27,28]. However, their cellular pathways and molecular mechanisms of action are still unknown.

Flavonoids are functional polyphenolic compounds widely distributed in natural sources, including medicinal plants and food sources [2,29,30]. Here, we report by means of liquid chromatography–tandem mass spectrometry the phenolic content of *P. sacharosa* leaves and show the presence of flavones, flavonones, and flavonol glycosides, as well as phenolic acids.

Some plant-derived metabolites can cross the BBB and exert protective effects against damage on neurons, astrocytes, microglia, and oligodendrocytes to protect these brain cells from damage, stimuli, or neurological deficits [2,31]. In agreement with our previous report [11], we observed self-fluorescence particles across the BBB of animals receiving EEPs or the fraction F10, lining along the lateral walls of the third ventricle and the hypothalamic arcuate nucleus of mice. Self-fluorescence was reminiscent of that of the kaempferol standard reported by Ruiz-Velasco-Martínez et al. [11] and the quercetin standard reported by Quinto-Ortiz et al. [32], indicating that both glycosylated and non-glycosylated forms of bioactive compounds can cross the BBB.

Emission spectra for kaempferol, quercetin, and myricetin have been reported using spectrofluorimetry in the range of 460–600 nm, using pH values above 10 [33,34]. Accordingly, optimal excitations of large clusters of flavonoid-like particles in F10 were observed between 425 and 525 nm, while abundant small flavonoid-like particles showed a wide spectrum from 425 to 645 nm. Self-fluorescence in EEPs and F10 was exclusively observed in thawed brain tissue, allowing us to track the bioactive self-fluorescent compounds in the interphase between the endothelial cells composing the BBB and the ependymal cells lining the walls of the third ventricle. Although large clusters of flavonoid-like particles were observed exclusively near the circumventricular organs and the cerebral ventricles, some could have been metabolized into smaller particles and diffused across the brain parenchyma to trigger or modulate the activity of specific groups of hypothalamic neurons located in nearby tissues, such as those from the arcuate nucleus. Indeed, the presence of flavonoids and structurally related metabolites has been reported in homogenates of rat cerebral tissue by chromatography and mass spectrometric approaches [35].

The arcuate nucleus is a key site that mediates leptin’s energy balance effects on glucose homeostasis through two neuronal populations: the anorexigenic appetite-suppressing pro-opiomelanocortin (POMC) neurons and the orexigenic appetite-increasing neuropeptide Y (NPY)/agouti-related peptide (AgRP) neurons [36]. Under negative energy balance conditions like those elicited by the peripheral administration of LPS, a reduction in food intake is commonly observed, followed by a fall in arc POMC and arc NPY/AgRP neuronal activity [37], in parallel to a decreased circulating thyroid hormone level, and an LPS-induced rise in corticosterone [17,38]. These hormonal signals are key elements to reduce energy expenditure and activate the immune response, triggered by the activation of a Toll-like receptor-4 (TLR4), a CD14 receptor for LPS, and the rise in pro-inflammatory cytokines such as IL-1β, IL-6, and TNF-α, which are essential for the induction of the inflammatory response [39,40,41,42,43]. Fasting increases the expression of the neuronal activity marker c-fos in NPY neurons [44,45] and reduces food intake due to LPS illness, increasing the number of cells expressing c-fos+, CD14+, or c-fos+/CD14+ mRNA, in a sub-population of the arcuate no larger than 20%, reminiscent of the total number of NPY cells in the arcuate [15].

Flavonoids such as kaempferol are known to attenuate LPS-induced neuro-inflammation by inhibiting the expression of several pro-inflammatory cytokines and BBB disruption [29,30]. In our experiments, CD14+ or c-fos+/CD14+ mRNA-positive cells were inhibited in animals who received EEPs 30 min before receiving LPS, similar to the expected response at the time point of 9 h, when pro-inflammatory signals have decreased, and anti-inflammatory signals are active to contain the inflammatory processes. The presence of glycosidic receptors in the arcuate nucleus that accept compounds as kaempferol when it is glycosylated, i.e., kaempferol-3-O-β-rutinoside [46,47], could have played a determinant role in the defense against microbial LPS.

The food intake hormonal signals are accompanied by excitatory and inhibitory neurotransmission systems, which modulate the electrical activity in the brain. Thus, the orexigenic neurons of the arcuate synthesize the neurotransmitter GABA in addition to NPY/AgRP, thereby inhibiting the response of anorexigenic neurons of the same arcuate in a paracrine way [48] or through second-order glutamatergic-anorexigenic neurons via direct afferents in the hypothalamus [49,50]. To measure the electrical activity of the arcuate in mice that received EEPs or F10 prior to LPS administration, we inserted an electrode in both hemispheres of the brain. Since the percentage of survival after surgery was low and it was not possible to carry out a statistical analysis of the groups, we proceeded to measure the electrical activity in the brains of rats. It has been reported that the administration of similar doses of LPS activates the IL-1β and IL-6 proinflammatory response in the periphery during the first 3–6 h, in parallel with a decrease in the expression of thyroid hormone transporters in the BBB and a decrease in food intake due to the activation of IL-1 receptors in the arcuate, both in C57/BL6 mice and Sprague Dawley rats [13,14,15].

The increased activity of chemokines, cytokines, GABA, and glutamate elements by LPS treatment is observed in many brain regions [51,52]. In the hippocampus, the increase in GABAergic synapses induced by LPS-mediated microglial activation leads to cognitive dysfunction, which is reversed 24 h after treatment [52]. Moreover, the acute or chronic administration of LPS modifies the GABAergic tone and provokes changes in intrinsic properties of the membrane in hippocampal CA1 neurons [53,54], which may lead to a decrease in theta oscillation in rats [55]. We demonstrated that an acute dose of LPS is enough to decrease theta oscillations in the hippocampus of freely moving rats and that the pretreatment with F10, 30 min before LPS administration, avoids the fall in theta oscillation provoked by LPS in the hippocampus, indicating that F10 can decrease the signs of inflammation at early times such as 3 h of LPS, saving the animal 6 h of discomfort and allowing the early recovery of the animal. Our results suggest that theta oscillations in the hippocampus may depend on the GABAergic tone since F10 induces a decrease in GABA_A_ γ2 receptor activity; however, the involved pathways are unknown.

The peripheral rise in corticosterone in the first 3 h of LPS exposure [17] promote the increase in IL-1β, IL-6, and TNF-α, exerting an anxiety effect on the limbic system, including the hippocampus [56]. Since the GABAergic inhibitory transmission modulates anxiety-related behavior and F10 down-regulated GABA_A_ γ2 receptor mRNA expression, it is feasible that flavonoids such as kaempferol or quercetin could have bound with greater affinity to the benzodiazepine (BZD) site of GABA_A_ receptors, promoting anxiolytic effects.

On the other hand, a reduction in food intake is observed after LPS administration. Contrary to fasting, LPS induces an inhibitory effect on arcuate NPY orexigenic neurons when glucose levels decline, by increasing IL-1β receptor expression [15] and TNF-α [37] in NPY neurons. Since orexigenic NPY neurons co-express GABA and anorexigenic POMC neurons express GABA_A_ receptors, an interplay between NPY/GABA- POMC/GABA_A_ receptors might exist in the hypothalamic arcuate of the animals that received F10 before LPS, due to high levels of quercetin-3-galactoside which may alter the glucose sensitivity of NPY/AgRP neurons and energy homeostasis.

Together, these findings support the hypothesis that phenolic components of EEPs modulate the GABAergic activity in the brain [57]. Although traditional practices in alternative medicine suggest the application of *P. sacharosa* for pain relief, and our evidence indicates positive results in reducing the central response to inflammation, further studies are required to assess the potential long-term side-effects of *P. sacharosa* extracts. It is relevant to note that toxicity studies with a lethal dose (LD)-50 have demonstrated that the methanolic extract of closely related plant species does not present cytotoxicity in healthy mice [58]. We used the ethanolic extract instead of the methanolic extract to prevent traces of the solvent from exhibiting an adverse effect that would mask the *P. sacharosa* effects. Since the highest dose administered in *Pereskia*-related plants was eighty-three times greater than the dose used in this study [58], and it does not show evidence of adverse effect, its consumption should be safe for humans and animals.

## 5. Conclusions

We conclude that bioactive compounds of *P. sacharosa* leaves cross the blood–brain barrier and reduce the central response to inflammation, allowing the early return of the ambulatory activity and well-being of the animal as demonstrated by the decreased expression of c-fos and CD14 mRNA in the arcuate, in addition to that for the GABA_A_ γ2 receptor in the hippocampus and the prevention of the fall in theta oscillations provoked by LPS. Although traditional practices in alternative medicine suggest the application of *P. sacharosa* for pain relief, and our evidence indicates positive results to reduce the central response to inflammation, further studies are required to assess the potential long-term side-effects of *P. sacharosa* extracts.

## Figures and Tables

**Table 1:** Phenolic acids and flavonoids (ng/g) in the *P. sacharosa* leaves.

Phenolic Metabolites		Concentration (ng/g)
Flavonoids		
	Apigenin	nd
	Apigeninidin	nd
	Catechin	nd
	Daidzein	nd
	Delphinidin	nd
	Epicatechin	nd
	Genistein	nd
	Hesperetin	nd
	Kaempferol	66.69
	Luteolin	nd
	Naringenin + Naringenin chalcone	4.50
	Proanthocyanidin A2	nd
	Procyanidin B2	nd
	Phloretin	nd
	Quercetin	152.89
	Quercetin-3-galactoside	482.88
	Quercetin-3-glucoside	5.49
	Resveratrol	nd
	Rutin	nd
Phenolic acid		
	Caffeic acid	564.78
	Chlorogenic acid	271.02
	Cinnamic acid	2803.64
	*p*-Coumaric acid	2637.72
	Ferulic acid	1609.37
	Gallic acid	9.92
	Protocatechuic acid	nd
	Syringic acid	388.83
	Vanillic acid	1237.25

nd: not detected.

## Data Availability

Data are contained within the article.

## Abbreviations

AgRP	Agouti-related peptide
BP	Bandpass
BBB	Blood–brain barrier
BW	Body weight
CNS	Central nervous system
DM	Dichromatic mirror
Dig	Digoxigenin
DTT	Dithiothreitol
DW	Dry weight
EEPs	Ethanolic extract of *P. sacharosa*
FFT	Fast Fourier Transform
F10	Flavonoid fraction
FISH	Fluorescent in situ
GABA_A_ γ2	GABA A gamma 2 receptor subunit
IOD	Integrated optical density
ISS	Isotonic saline solution
L-Hip	Left hippocampus
LC-MS/MS	Liquid chromatography–tandem mass spectrometry
LFP	Local field potential recording
LP	Low pass
LPS	Bacterial lipopolysaccharide
MBH	Mediobasal hypothalamus
NPY	Neuropeptide Y
PEG	Polyethylene glycol
POMC	Pro-opiomelanocortin
R-Hip	Right hippocampus
SDS	Sodium dodecyl sulfate
SSB	Stainless steel beads
SSC	Standard saline citrate
TLC	Thin-layer chromatography
TLR4	Toll-like receptor-4
TSA	Tyramide Signal Amplification
UV	Ultraviolet light
